# Association between built environments and weight status: evidence from longitudinal data of 9589 Australian children

**DOI:** 10.1038/s41366-022-01148-6

**Published:** 2022-05-30

**Authors:** I Gusti Ngurah Edi Putra, Thomas Astell-Burt, Xiaoqi Feng

**Affiliations:** 1 Population Wellbeing and Environment Research Lab (PowerLab), Wollonggong, NSW Australia; 2 School of Health and Society, Faculty of the Arts, Social Sciences and Humanities, University of Wollongong, Wollongong, NSW Australia; 3 School of Population Health, Faculty of Medicine and Health, University of New South Wales, Sydney, NSW Australia

**Keywords:** Risk factors, Epidemiology, Epidemiology

## Abstract

**Background:**

No studies appear to examine potential associations between changes in built environments across childhood and the developmental trajectories of child weight status.

**Objective:**

Examine the developmental trajectories of child weight status with respect to changes in childhood exposure to the built environments.

**Methods:**

This study used data of 9589 children with biennial follow-up (2004–2016), retrieved from the Longitudinal Study of Australian Children. Changes in objectively-measured child weight status (i.e., body mass index-BMI, waist circumference) were investigated in relation to changes in seven built environments (i.e., neighbourhood safety; green space quality; footpaths and street conditions; public transport; shopping facilities; basic services; and local traffic) subjectively reported by caregivers. Group-based discrete trajectory mixture models were used to classify children according to their developmental trajectories of built environments and weight status. Multilevel multinomial logistic regression was employed to examine associations between built environments and child weight status adjusted for confounding.

**Results:**

Two, four, and six trajectory groups were developed for built environment variables. Three groups namely “moderate”, “high”, and “extreme increase” were generated for each BMI and waist circumference. Findings from multilevel analyses indicated that growing up in neighbourhoods that are considered highly safe, with better quality of green space nearby, and in areas with low local traffic over time are protective against unhealthy weight increase in childhood. Meanwhile, living with better access to shopping facilities and basic services was associated with an unhealthy increase in BMI and/or waist circumference. No clear associations appeared between the quality of footpath and street conditions, access to public transport, and child weight status.

**Conclusions:**

Built environments might act either as a risk or protective factor of an unhealthy increase in child weight status. Enabling health-promoting neighbourhoods (i.e., highly safe, quality green space nearby, low local traffic) is important to support a healthy weight trajectory across childhood.

## Introduction

Childhood obesity is one of the most significant public health concerns of the 21^st^ century [1]. The World Health Organisation (WHO) reported that the global prevalence of overweight and obesity among children and adolescents (5–19 years) increased from 4% in 1975 to more than 18% in 2016 [2]. The prevalence of overweight and obesity among children and adolescents in developed countries is much higher at about 23.8% and 22.6% for boys and girls, respectively and as compared with 12.9% and 13.4% for boys and girls in developing countries in 2013 [3]. In Australia, one in four (25%) children and adolescents (5–17 years) were overweight or obese in 2017–2018 and 8.2% of them were obese [4]. The translation of child obesity into adult obesity is socioeconomically patterned, with higher levels in disadvantaged communities [5]. In addition, findings from biennial follow-up of the same children in the Longitudinal Study of Australian Children (LSAC) suggest that the prevalence of overweight and obesity increased with age [4, 6].

There has been growing interest in understanding the potential roles of built environments in influencing child weight-related outcomes. Evidence from previous systematic reviews indicates that built environments, including access to green space [7, 8] and access to neighbourhood sidewalks [9] are associated with lower body mass index (BMI) and/or the decreased odds of unhealthy weight (i.e., overweight or obese) among children. However, no clear conclusions appear on the influence of other types of built environments in relation to the risk of overweight or obesity among children, such as access to public transport [10] and grocery stores [11].

Some mechanisms might explain the built environment—child weight association. Built environments might lead to healthy weight among children through health-promoting behaviours. For example, physical activity and/or sedentariness might serve as a pathway in which neighbourhood green space [12, 13], safety [14, 15], neighbourhood sidewalk [9], and local traffic [16] influence child weight status. By reducing harmful environmental stressors, such as air pollution, children benefit from living in greener [17, 18] and low-traffic neighbourhoods [19] in relation to their healthy body weight. Moreover, the improvement in a positive emotional state might explain why exposure to neighbourhood green space [20, 21] and safety [22] are important to prevent children gain excess weight.

To our knowledge, some longitudinal studies have been designed based on the assumption that built environments remain stable over time [23–25], ignoring potential influences of changes in built environments that may influence changes in health and related outcomes [26]. For example, while there is a legion of linking green space with health, longitudinal evidence linking green space qualities and health remains volumetrically smaller [27] and, but for some recent studies [28, 29], rarely examines how subjective and or objective changes in those qualities may lead to changes in health status [30]. This is a widespread issue in the built environment and health literature as reported in a scoping review [31], which summarised a number of studies that measured changes (i.e., developmental trajectories) in weight status (e.g., BMI), including child and adolescent participants, but none of these studies evaluated changes in built environments as exposure of interest. This present study aimed to enrich the current literature by examining the associations between changes in a range of built environments and developmental trajectories of weight status among Australian children from age 2 to 15 years.

## Methods

### Data

This study used data from the nationally representative cohort study, *Growing Up in Australia*: the Longitudinal Study of Australian Children (LSAC) [32]. Two-stage clustered probability sampling was used to recruit children. In the first step, postcodes where children lived, were selected using the probability proportional to size technique considering geographical stratifications by state, capital city vs. the rest of state area, and urban-rural status. Children and their caregivers were then recruited from a representative sample of postcodes. Detailed information on the LSAC’s content and methodology can be found elsewhere [33, 34]. This present study involved data from 9589 children that consisted of 4606 children from “baby” (B) cohort Waves 2 to 7 (children aged 2–3 years to 12–13 years; 2006 to 2016) and 4983 children from “kindergarten” (K) cohort Waves 1 to 6 (aged 4–5 years to 14–15 years; 2004 to 2014). The selection of aforementioned waves in respective cohorts due to both data of caregiver-perceived built environments and weight status (BMI and waist circumference) were consistently collected. The LSAC has obtained ethics approval from the Australian Institute of Family Studies Ethics Committee.

### Perceived built environments

We used caregiver-based biennial assessments on seven aspects of built environments, namely neighbourhood safety; green space; footpaths, roads, and street lighting; public transport; basic shopping facilities; basic services; and heavy traffic (Table S1). These variables have been used to study neighbourhood influences on child and adult health and weight status within the Australian context [28, 29, 35–40]. Caregiver responses from strongly disagree to strongly agree for each built environment measure were dichotomised into disagree (for “strongly disagree” and “disagree”) and agree (“for agree” and “strongly agree”) to enable the discrete trajectory mixture models (see ‘Data Analysis’ below).

### Child weight status

BMI and waist circumference were biennially measured by a trained interviewer. Weight in light clothing was measured to the nearest 50 g using glass bathroom scales (Salter Australia; Code 79985); and height to the nearest 0.1 cm was assessed using a portable rigid stadiometer (Invicta; Code IPO955) [23]. Waist circumference was measured to the nearest 0.1 cm horizontally around the navel using a non-stretch dressmaker’s tape. The interviewer took twice the height and waist circumference measures and then calculated the average. When the first and second measures were different by more than 0.5 cm, a third one was taken and the average was calculated based on the two closest measures [41]. BMI (in kg/m^2^) and waist circumference (in cm) were then classified into some groups using the discrete trajectory mixture models (see ‘Data Analysis’ below).

### Confounders

Confounders were selected based on a conceptualisation that they might influence neighbourhood selection [42, 43]. These variables were also treated as confounders in previous studies on neighbourhood environments and child health outcomes [23, 36, 44]. Children’s age, sex, Indigenous status, and whether children spoke a language other than English at home were variables representing individual characteristics. Family socioeconomic characteristics encompassed the highest educational level of a caregiver in the family, a combined weekly income of both caregivers, family structure, and the number of siblings the study child had. In addition, two neighbourhood measures were included. Area disadvantage was assessed using the Index of Relative Socioeconomic Disadvantage from the Socio-economic Indexes for Areas (SEIFA) [45] and then classified into tertiles. Area accessibility was determined using the Accessibility-Remoteness Index of Australia (ARIA) [46].

### Data analysis

Group-based discrete trajectory mixture models were employed to develop trajectories of built environments, BMI, and waist circumference across the period of both cohorts combined. This has been used by previous studies in modelling the trajectories of BMI [31] and mental health [47]. Group-based discrete trajectory mixture models partition individuals into some groups, denoting differences in trajectory courses. This method identifies distinctive clusters of individuals following similarly developmental trajectory measures of an outcome (e.g., health outcome or behaviour) over time. Maximum likelihood is used to estimate the model parameters [48, 49]. We used “Traj” macro in STATA V14.2 to conduct the analysis, guided by previous literature [50]. Binary logit and censored normal distributions were used to fit trajectory models for perceived built environments (dichotomous variables) and weight status (continuous variables), respectively. The optimal number of trajectory groups was selected based on the lowest Bayesian Information Criterion (BIC) values with a minimum group size of 5% of the total participants [51, 52]. Table S2 presents the BIC values of trajectory groups.

Following the identification of trajectory groups, we examined factors associated with trajectory group membership and confounders-adjusted associations between built environments and weight status. In LSAC, children’s observations were collected multiple times throughout time that were nested within individuals. Therefore, we used multilevel regression models for repeated-measure structured hierarchical data [53], taking into account clustering effects [54, 55] and correlated observations [56, 57]. Three-level multinomial logistic regression was fitted in MLwIN V3.01 [58] with observations across waves (level 1) that were nested within children (level 2) and statistical areas, level 2 (SA2s) (level 3). SA2s are standard geographical areas available in LSAC, classifying some suburbans within cities and outside city areas where communities with around 10 000 residents can interact socially and economically [59]. Findings from this analysis were reported as adjusted relative-risk ratio (RRR) along with 95% confidence intervals (CIs).

## Results

Table 1 presents the baseline characteristics of the samples. Balanced proportions between boys and girls were recruited. The proportion of Indigenous children was up to 5% and no more than 14% of children spoke a language other than English at home. The majority of caregivers in the family completed above high school education. On average, caregivers at the baseline in the B cohort and the K cohort earned 1470 and 1270 AUD on weekly basis, respectively. Most of the children lived with two caregivers at home and had one to two siblings. Children were almost proportionately distributed in high, moderate, and low disadvantaged areas and more than half resided in highly accessible areas.

**Table 1:** Baseline characteristics of children.

Variables	B cohort Wave 2 (2–3 years) *n* (%^a^)	K cohort Wave 1 (4–5 years) *n* (%^a^)
**Number of children**	4606	4983
**Children characteristics**		
Child’s sex		
Female	2257 (48.92)	2447 (48.77)
Male	2349 (51.08)	2536 (51.23)
Child Indigenous status		
Non-Indigenous	4426 (94.88)	4794 (96.07)
Indigenous	180 (5.12)	187 (3.90)
* missing/not reported*		2 (0.03)
Child speaks a language other than English		
No	4150 (87.80)	4359 (86.00)
Yes	453 (12.14)	624 (14.00)
* missing/not reported*	3 (0.06)	
**Family characteristics**		
Caregiver education		
≤High school	592 (15.55)	923 (20.38)
>High school	4014 (84.45)	4056 (79.56)
*missing/not reported*		4 (0.06)
Family weekly income (in thousands), mean (SD)	1.47 (1.15)	1.27 (0.86)
Family structure		
One-caregiver family	507 (13.25)	697 (14.95)
Two-caregiver family	4099 (86.75)	4286 (85.05)
Number of siblings, mean (SD)	1.29 (1.07)	1.51 (1.07)
**Neighbourhood characteristics**		
Area disadvantage (SEIFA)		
High	1623 (38.16)	1794 (37.24)
Moderate	1591 (33.79)	1611 (32.89)
Low	1392 (28.05)	1578 (29.87)
Area accessibility (ARIA)		
Highly accessible	2464 (55.17)	2702 (55.35)
Accessible	1143 (24.08)	1163 (24.05)
Moderately accessible	765 (16.27)	856 (16.09)
Remote	109 (2.06)	126 (2.02)
Very remote	70 (1.53)	90 (1.77)
* missing/not reported*	55 (0.90)	46 (0.73)

^a^weighted percentage.

### Trajectory groups of perceived built environments and factors associated with group membership

**Fig. 1:**
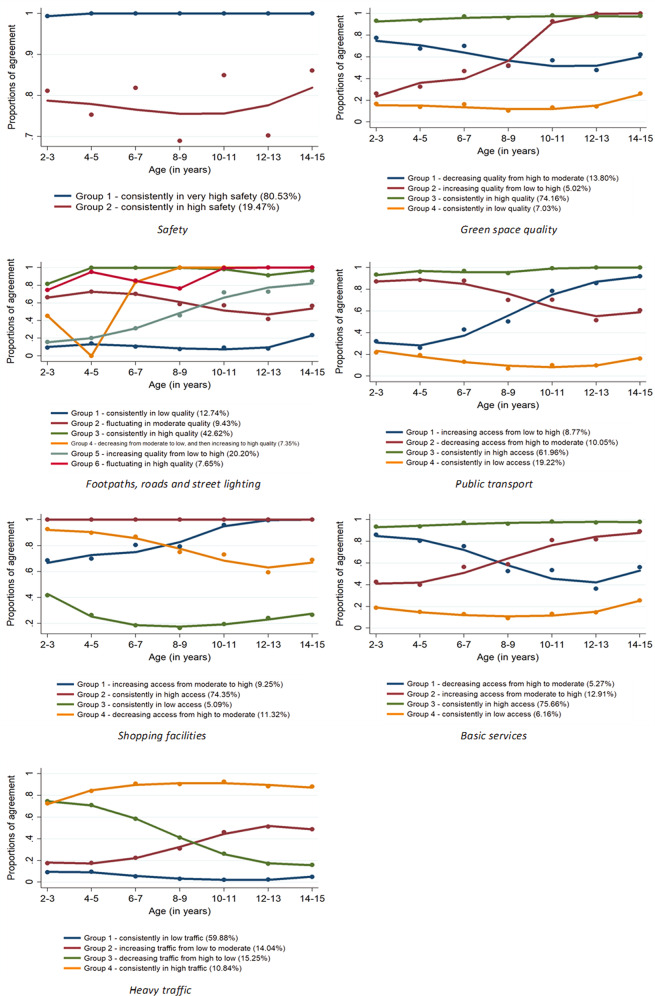
Trajectory groups of caregiver-perceived built environments among 9589 Australian children.

Some trajectory groups were developed for each built environment, representing distinctive clusters of changes as children became older. Two and six groups were developed for neighbourhood safety; and footpaths, roads, and street lighting aspects; respectively. Four classes were generated for other built environment variables such as green space quality; public transport; shopping facilities; basic services; and heavy traffic, considering BIC values and none of the groups with less than 5% of the total respondents (Table S2). The percentages of individuals who occupied each trajectory group are presented in Fig. 1. Overall, children were partitioned into groups where caregivers reported consistently high or low access to, or quality of, built environments throughout the cohort. Also, other groups of children whose caregivers perceived that the access to, or quality of, built environments increased or decreased over time.

Factors associated with trajectory group membership for built environment variables are presented in Tables S3.1 to S3.7. Neighbourhood characteristics such as area disadvantage and area accessibility were consistently associated with trajectory group membership. Living in more affluent areas was associated with membership in trajectory groups with higher neighbourhood safety, favourable green space quality, better quality of footpaths, roads, and street lighting, higher access to public transport, shopping facilities, and basic services, but groups with heavy local traffic. Living in more remote areas was found to reduce the likelihood of being in groups with better surrounding green space quality, better footpaths, roads and street lighting quality, higher access to public transport, shopping facilities, and basic services, and higher local traffic. Meanwhile, children residing in accessible and moderately accessible areas were more likely to be in a group whose caregivers reported highly safe neighbourhoods compared to those who lived in highly accessible areas. No differences in the likelihood of living in highly safe neighbourhoods between those who were from highly accessible and remote areas. Furthermore, children and family socio-demographic characteristics were not consistently associated with trajectory group membership for all built environment variables.

### Trajectory groups of weight status and factors associated with group membership

**Fig. 2:**
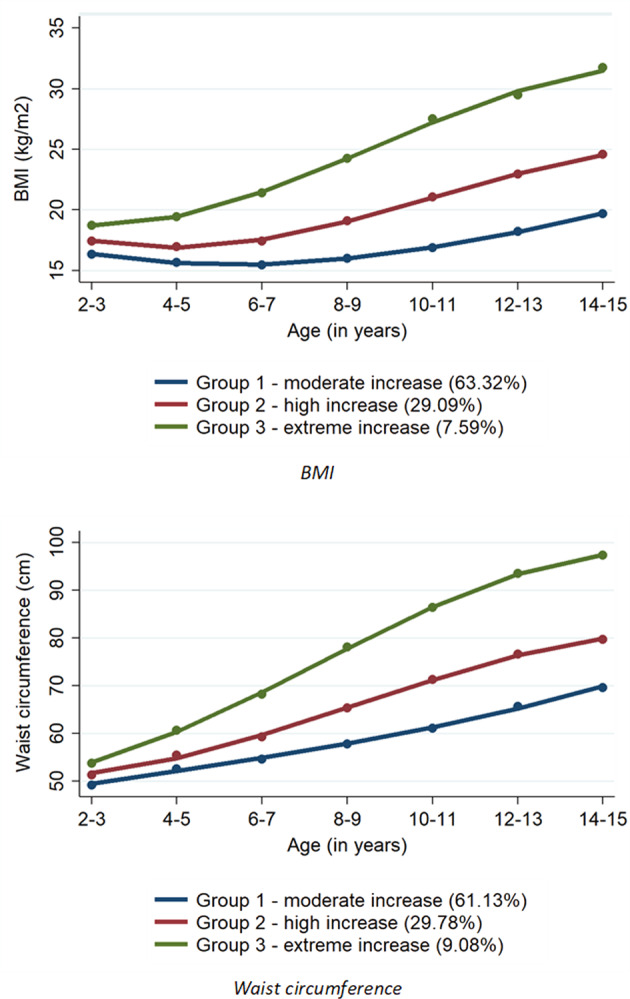
Trajectory groups of BMI and waist circumference among 9589 Australian children.

Three similar trajectory groups were developed for weight status, namely moderate, high, and extreme increases in BMI or waist circumference (Fig. 2). For both weight status measures, more than half of children belonged to a group of moderate increase, followed by high and extreme increase groups, accounting for less than 30% and 10%, respectively. Table 2 presents factors associated with membership of trajectory groups of weight status. Older age was associated with the increased likelihood of being in groups with high and extreme increases, relative to moderate increase in weight status. While boys were less likely to have high and extreme increases in BMI, they had a higher likelihood of high and extreme increases in waist circumference compared to girls. Non-Indigenous children were less likely to have an extreme increase in weight status than their Indigenous counterparts. Children who only spoke English at home decreased the likelihood of an extreme increase in weight status. Favourable family socioeconomic status such as better educational level of caregivers or higher household income was negatively associated with being in groups with high or extreme increases in weight status. Living with two caregivers at home was also protective against experiencing an unhealthy weight increase. In addition, a lower likelihood of having high or extreme increases in BMI and waist circumference was found among children living in less disadvantaged areas. However, no association was apparent between area accessibility and trajectory group membership of weight status.

**Table 2:** Associations between socio-demographic characteristics and trajectory groups of weight status.

Variables	BMI		Waist circumference	
	Group 2–high increase RRR (95% CI)	Group 3–extreme increase RRR (95% CI)	Group 2–high increase RRR (95% CI)	Group 3–extreme increase RRR (95% CI)
	*Reference group: Group 1 – moderate increase*		*Reference group: Group 1–moderate increase*	
**Child characteristics**				
Child’s age *(ref:* 2,3 *year)*				
4–5 years	1.02 (0.94; 1.11)	1.08 (0.93; 0.26)	1.04 (0.96; 1.14)	1.12 (0.97; 1.29)
6–7 years	1.06 (0.97; 1.16)	1.12 (0.96; 1.31)	1.09 (1.00; 1.18)	**1.16 (1.01; 1.34)**
8–9 years	1.09 (0.99; 1.19)	1.17 (1.00; 1.37)	**1.11 (1.01; 1.21)**	**1.22 (1.05; 1.41)**
10–11 years	1.09 (1.00; 1.20)	1.18 (1.00; 1.39)	**1.12 (1.02; 1.22)**	**1.23 (1.05; 1.43)**
12–13 years	1.10 (1.00; 1.22)	**1.22 (1.03; 1.45)**	**1.13 (1.02; 1.24)**	**1.27 (1.08; 1.49)**
14–15 years	1.09 (0.97; 1.22)	1.20 (0.98; 1.47)	1.10 (0.99; 1.23)	**1.27 (1.05; 1.52)**
Child’s sex *(ref: Female)*				
Male	**0.80 (0.75; 0.86)**	**0.85 (0.76; 0.96)**	**1.17 (1.09; 1.24)**	**1.47 (1.32; 1.64)**
Child Indigenous status *(ref: Indigenous)*				
Non-Indigenous	1.04 (0.87; 1.25)	**0.69 (0.51; 0.93)**	1.11 (0.92; 1.34)	**0.63 (0.48; 0.83)**
Child speaks a language other than English *(ref: Yes)*				
No	**0.88 (0.79; 0.98)**	**0.56 (0.47; 0.68)**	0.97 (0.86; 1.08)	**0.64 (0.54; 0.76)**
**Family characteristics**				
Caregiver education *(ref:* ≤ High school)				
>High school	1.01 (0.92; 1.12)	**0.82 (0.70; 0.97)**	0.97 (0.88; 1.06)	0.88 (0.76; 1.02)
Family weekly income (in thousands)	**0.97 (0.95; 0.99)**	**0.85 (0.81; 0.89)**	0.98 (0.96; 1.01)	**0.86 (0.82; 0.89)**
Family structure *(ref: One-caregiver family)*				
Two-caregiver family	**0.91 (0.83; 0.99)**	0.90 (0.78; 1.04)	**0.91 (0.83; 0.99)**	**0.86 (0.75; 0.98)**
Number of siblings, mean (SD)	**0.97 (0.94; 0.99)**	0.98 (0.93; 1.03)	**0.95 (0.93; 0.98)**	0.97 (0.92; 1.01)
**Neighbourhood characteristics**				
Area disadvantage (SEIFA) *(ref: High)*				
Moderate	**0.92 (0.86; 0.98)**	**0.80 (0.71; 0.89)**	0.95 (0.89; 1.01)	**0.84 (0.76; 0.93)**
Low	**0.83 (0.77; 0.90)**	**0.63 (0.54; 0.72)**	**0.89 (0.82; 0.96)**	**0.68 (0.60; 0.77)**
Area accessibility (ARIA) *(ref: Highly accessible)*				
Accessible	0.99 (0.91; 1.07)	1.09 (0.95; 1.26)	0.98 (0.91; 1.06)	1.12 (0.98; 1.28)
Moderately accessible	0.97 (0.89; 1.07)	1.00 (0.84; 1.18)	0.99 (0.90; 1.09)	1.14 (0.98; 1.33)
Remote	0.94 (0.77; 1.17)	1.29 (0.90; 1.83)	0.94 (0.76; 1.16)	1.23 (0.88; 1.72)
Very remote	0.97 (0.75; 1.25)	0.90 (0.56; 1.44)	0.94 (0.72; 1.21)	1.09 (0.71; 1.66)

*RRR* = adjusted relative-risk ratio, *CI* = confidence interval, bold = *p*-value < 0.05.

### Built environments and weight status

Table 3 presents confounders-adjusted associations between each built environment and weight status. Relative to moderate increase, children consistently living in very highly safe neighbourhoods were less likely to be in groups with high and extreme increases in BMI and waist circumference compared to those in safe neighbourhoods. Children whose caregivers reported that green space quality increased from low to high or was consistently in high quality were less likely to have an extreme increase in BMI and waist circumference compared to those whose caregivers perceived neighbourhood green space in low quality over time. Meanwhile, decreasing the quality of green space from high to moderate was associated with being in a group with a high increase in waist circumference. The quality of footpaths, roads, and street lighting was not associated with BMI. However, caregiver perceptions of this built environment fluctuated in moderate or high quality were associated with an increased likelihood of being in groups with high and/or extreme increases in waist circumference.

**Table 3:** Adjusted associations between perceived built environments and weight status.

Variables	BMI		Waist circumference	
	Group 2—high increase RRR (95% CI)	Group 3—extreme increase RRR (95% CI)	Group 2—high increase RRR (95% CI)	Group 3—extreme increase RRR (95% CI)
	*Reference group: Group 1–moderate increase*		*Reference group: Group 1–moderate increase*	
**Built environment variables**				
Safety *(ref: Class 2*—*consistently in high safety)*				
Class 1—consistently in very high safety	**0.83 (0.76; 0.90)**	**0.71 (0.62; 0.82)**	**0.86 (0.79; 0.93)**	**0.73 (0.64; 0.83)**
Green space quality *(ref: Class 4*—*consistently in low quality)*				
Class 1—decreasing quality from high to moderate	1.15 (0.98; 1.35)	0.94 (0.72; 1.23)	**1.23 (1.05; 1.44)**	0.90 (0.70; 1.16)
Class 2—increasing quality from low to high	1.11 (0.92; 1.34)	**0.60 (0.43; 0.84)**	1.22 (1.00; 1.48)	**0.60 (0.44; 0.81)**
Class 3—consistently in high quality	1.01 (0.88; 1.17)	**0.75 (0.59; 0.95)**	1.14 (0.99; 1.32)	0.83 (0.67; 1.04)
Footpaths, roads and street lighting *(ref: Class 1 – consistently in low quality)*				
Class 2—fluctuating in moderate quality	1.09 (0.95; 1.25)	1.26 (0.99; 1.60)	**1.17 (1.02; 1.35)**	**1.32 (1.06; 1.64)**
Class 3—consistently in high quality	0.90 (0.80; 1.00)	0.84 (0.69; 1.02)	0.93 (0.84; 1.05)	0.85 (0.71; 1.02)
Class 4—decreasing quality from moderate to low, and then increasing to high quality	1.06 (0.91; 1.23)	0.79 (0.60; 1.03)	1.08 (0.93; 1.25)	0.94 (0.73; 1.21)
Class 5—increasing quality from low to high	1.04 (0.93; 1.18)	1.12 (0.91; 1.38)	1.03 (0.92; 1.17)	1.03 (0.85; 1.25)
Class 6—fluctuating in high quality	1.14 (0.98; 1.32)	0.95 (0.73; 1.23)	**1.36 (1.18; 1.58)**	1.20 (0.94; 1.53)
Public transport *(ref: Class 4 – consistently in low access)*				
Class 1—increasing access from low to high	1.01 (0.89; 1.15)	0.90 (0.72; 1.13)	1.02 (0.89; 1.15)	0.86 (0.70; 1.07)
Class 2—decreasing access from high to low	0.92 (0.81; 1.05)	**0.75 (0.60; 0.94)**	1.00 (0.88; 1.14)	**0.74 (0.60; 0.91)**
Class 3—consistently in high access	0.92 (0.84; 1.02)	1.05 (0.89; 1.24)	0.96 (0.87; 1.06)	0.91 (0.78; 1.07)
Shopping facilities *(ref: Class 3 – consistently in low access)*				
Class 1—increasing access from moderate to high	0.99 (0.82; 1.19)	**1.64 (1.17; 2.30)**	0.94 (0.78; 1.13)	**1.59 (1.18; 2.15)**
Class 2—consistently in high access	**0.84 (0.72; 0.99)**	**1.46 (1.08; 1.99)**	0.89 (0.76; 1.04)	1.23 (0.94; 1.60)
Class 4—decreasing access from high to moderate	0.97 (0.81; 1.15)	**1.60 (1.16; 2.22)**	0.97 (0.81; 1.15)	**1.49 (1.11; 2.00)**
Basic services *(ref: Class 4*—*consistently in low access)*				
Class 1—decreasing access from high to moderate	0.96 (0.79; 1.16)	1.06 (0.75; 1.51)	0.88 (0.73; 1.06)	0.91 (0.67; 1.25)
Class 2—increasing access from moderate to high	0.87 (0.74; 1.02)	**1.38 (1.02; 1.85)**	1.01 (0.86; 1.19)	1.22 (0.94; 1.60)
Class 3—consistently in high access	0.87 (0.75; 1.00)	**1.37 (1.05; 1.77)**	0.86 (0.75; 1.00)	1.12 (0.89; 1.42)
Heavy traffic *(ref: Class 1 – consistently in low traffic)*				
Class 2—increasing traffic from low to moderate	**1.22 (1.12; 1.34)**	**1.49 (1.27; 1.76)**	**1.17 (1.07; 1.28)**	**1.24 (1.07; 1.44)**
Class 3—decreasing traffic from high to low	**1.24 (1.13; 1.37)**	**1.42 (1.21; 1.68)**	**1.17 (1.07; 1.29)**	**1.43 (1.23; 1.67)**
Class 4—consistently in high traffic	**1.32 (1.19; 1.48)**	**1.35 (1.11; 1.64)**	1.10 (0.99; 1.23)	**1.34 (1.12; 1.59)**

*RRR* = adjusted relative-risk ratio, *CI* = confidence interval, bold = *p*-value < 0.05.

Separate models were developed for each built environment variable. The model was adjusted for child’s age, sex, Indigenous status, language spoken at home, caregiver education, family income, family structure, number of siblings, area disadvantage, and area accessibility.

Access to public transport that increased or decreased between low and high levels was associated with a lower likelihood of experiencing an extreme increase in weight status, though only decreasing access from high to low showed a statistically significant association. Children with better access to shopping facilities and basic services were more likely to be found with an extreme increase in BMI and/or waist circumference. Compared to those living in areas with low traffic, children growing up in areas with heavy local traffic were more likely to have high and extreme weight increases.

## Discussion

### Key findings

To our knowledge, this study is potentially the first that used group-based trajectory models to pattern changes in built environments across childhood and assess whether these changes matter for the developmental trajectories of BMI and waist circumference. Overall, findings indicate that associations between built environments and child weight status varied by the type of the built environments being tested. Favourable changes or stability in exposure to some built environments tended to be protective against unhealthy weight gain in childhood. For example, children living in perceived-safe neighbourhoods over time, and with green space reported, in high quality or, to increase from low to high quality, as they became older, were more likely to maintain healthy weight across childhood. However, better access to basic services (i.e., increasing from moderate to high, consistently in high access) was associated with an extreme increase in BMI. Children experiencing consistent access to shopping facilities at a high level, or changes in access, irrespective of direction or level (i.e., moderate to high or high to moderate), relative to low access, were more likely to experience an extreme increase in both BMI and waist circumference. In addition, changes in local traffic conditions from low to moderate, high to low, or stable in high traffic throughout time increased the likelihood of unhealthy weight increase. Associations between changes in footpaths, roads, and street lighting quality; access to public transport; and child weight status were unclear.

### Associations between changes in built environment and child weight and possible pathways

Caregiver perceptions of high neighbourhood safety over time were associated with healthy weight gain in childhood. Findings from a recent meta-analysis indicated that living in unsafe neighbourhoods is associated with a reduction in physical activity and an increase in BMI among children [60]. Unsafe neighbourhood perceived by parents might discourage outdoor activities among children, which in turn, can lead to sedentariness, such as having more screen time [14, 15]. Another pathway, such as mental health was also reported where biological stress indexed by telomere length serves as a pathway in which neighbourhood violence may influence childhood obesity [22].

Green space trended from low to high or was in high quality throughout time was less likely to be associated with an extreme increase in BMI and waist circumference as children aged. Mechanisms through encouraging physical activity and/or reducing screen time might help explain the association among children [12, 13]. Air pollution exposure might also serve as a pathway in this regard [17, 18]. Importantly, while most of the evidence on green space and child weight heavily relied on a quantitative measure of green space [7, 8], this study represented a step forward in the literature by investigating green space quality with changes in weight status among children. Caregiver-reported green space quality, relative to quantity (e.g., greenness), might serve as an important and more relevant measure of exposure to green space since children’s spatial mobilities are more likely to be determined by caregivers [35, 44].

Unfavourable local traffic conditions (i.e., increasing from low to moderate, consistently in high traffic) were associated with an unhealthy weight increase. Outdoor physical activity might also explain the association between high traffic and unhealthy weight trajectory [16]. Moreover, heavy traffic is also indicative of exposure to air-related pollution. Strong evidence on the association between exposure to air-related pollution, such as PM_2.5_, PM_2.5absorbance_, PM_10_, PM_coarse_, NOx, and NO_2_, and the risk of childhood obesity was reported in a recent meta-analysis [19]. Other pathways potentially linking air-related pollution to obesity include increasing blood pressure [61], total cholesterol and LDL levels [62], and poor sleep [63]. It is important to note that our study found that relative to consistently low traffic, *decreasing local traffic from high to low* was also associated with unhealthy weight gain. This might indicate that heavy local traffic at lower ages might provide adverse impacts on child weight even though the exposure to traffic decreases later in childhood. Young children might be the most vulnerable to the detrimental impacts of heavy traffic due to exposure to air pollution [64]. More empirical studies are needed to identify whether young ages are a sensitive period to the impacts of heavy traffic and its concomitants in relation to their weight status, as well as the possible pathways linking them.

Better access to basic services and shopping facilities (i.e., increasing from moderate to high, consistently in high access) increased the likelihood of an unhealthy weight trajectory. While high-to-moderate changes in access to shopping facilities might be expected to reduce the likelihood of unhealthy weight increase, a statistically significant association in the opposite direction appeared. The level of changes in the access (i.e., high to moderate) was certainly greater when it was compared to the low-level access stable in the reference group. This might explain why children with decreasing access to shopping facilities from high to moderate were more likely to experience an unhealthy weight increase, relative to their counterparts with consistently low access. A current review evaluating the association between the availability of grocery stores and child weight-related behaviours and outcomes suggested mixed findings that might be due to the variations in the study contexts [11]. In our study, the associations between access to shopping facilities, basic services, and unhealthy weight among children perhaps can be elucidated by obesogenic environmental factors, such as the proximity to fast-food restaurants and shops of sugar-sweetened beverages. Past work found that proximity to fast-food restaurants was associated with the increased risk of overweight or obesity [65], but the presence of more healthy food outlets nearby was associated with overweight in the opposite direction [66].

No clear association appeared between caregiver-perceived quality of footpaths, roads, and street lighting and objective measures of weight status. A possible explanation is that different elements of built environments (i.e., footpaths, roads, street lighting) were combined in a single question. Caregivers might unequally weight each element based on which they viewed as relatively more important before reaching an overall conclusion. More variety in caregivers’ responses to this question might elucidate why more trajectory groups were found to fit with the data compared to other built environment indicators. Nevertheless, previous studies indicated that better access to sidewalks can contribute to neighbourhood walkability which might play important roles in improving active commuting and reducing sedentary behaviour and the risk of overweight or obesity [9, 67]. The presence of better street lighting was found to be associated with traffic safety [68], which in turn, might lead to more physical activity [69].

Changes in caregiver perceptions of access to public transport that increased or decreased between low and high levels were associated with lower likelihood of the extreme increase in weight status among children, but only the perception decreasing from high to low showed a statistically significant association in this regard. A previous review reported heterogeneous findings on the association between access to public transport and childhood obesity [10]. Public transport includes various types of transportation modes and also encompasses active transport, public transit, and urban transit. Mixed findings from previous studies might suggest the strong influence of context-related factors. Even though public transport might be accessible, one’s decision to use public transport might also be determined by other factors, such as personal preferences, travel cost, the time allowed for travel, the availability of sidewalks, weather conditions, etc [10].

### Strengths and limitations

This study might be among the first that investigated changes in different built environments in relation to changes in weight status (i.e., BMI and waist circumference) across childhood. Findings from this study enrich the current literature on the importance of the accumulation of exposure to certain built environments. The use of longitudinal data in this study can provide better support for causality compared to previous studies that are cross-sectional in design. Findings from this study can be considered robust by using multilevel models and controlling all confounders, including neighbourhood characteristics that were strongly associated with built environment variables. Same-source bias might be not a concern in this study since the exposure and the outcome were derived from different sources, which may otherwise be a problem when reliant upon subjective reports of weight status that might not correlate particularly well with objective measures, while also permitting contrasting associations with health-related behaviors [70].

Limitations of this study included the measure of built environments. This study used subjective measures of built environments reported by caregivers due to the unavailability of objective measures. Changes reported by caregivers across childhood might not be fully indicative of actual changes in built environments since their perceptions can be influenced by personal values, norms, and socioeconomic background [21, 35]. This does not mean those perceptions should be dismissed as unimportant; caregiver-based subjective measures might be more relevant since they have experience of living in the neighbourhood on daily basis and tend to regulate their children’s outdoor activities. Those perceptions are likely to be informed by things privy to the caregiver that go unmeasured by geographic information systems and remotely sensed objective measures. It is therefore of some debate as to whether this constitutes a strength or limitation of the study at that level. However, there is limitation on the wording of the items that measure built environments. For example, the statement used to measure green space quality might include quantitative (i.e., there are parks, playgrounds and play spaces) and qualitative dimensions (i.e., good parks, playgrounds, and play spaces), but currently does not provide such nuance. To elaborate, caregivers whose responses were “disagree” will conflate those who perceived none of the aforementioned types of green space available in their neighbourhood and those who reported green space of low quality when any of them was available. Moreover, a measure of neighbourhood walkability combined three different aspects in a single item, such as footpaths, roads, and street lighting. It is understandable that such economies are often taken in large surveys that cover a huge range of issues, but it nonetheless means in this case that we cannot discern if the perception is weighted more by footpaths, roads, streetlight, or a combination of the three. This might also be a reason why no clear association appeared between this built environment in relation to child weight status.

The sample was predominantly from high-educated caregivers, two-caregiver families, non-Indigenous, and English-speaking children. The small proportion of some sub-sample groups can indicate less variability in the data that can influence the findings and their generalisability to some extent. This study also did not investigate the effect modifiers the reported associations, indicating further investigation is needed. This is particularly important from an equity perspective, not only based on evidence that communities living with socioeconomic disadvantage may benefit disproportionately more than their more advantaged counterparts from investments in built environment [71], but also given evidence of differences in associations reported in low and middle income countries [72, 73] sex and gender identity [74], and even personality traits such as extraversion and neuroticism [75]. That research is crucial to ensure that recommendations for urban planning result in more inclusive and equitable cities that promote health across their diverse populations.

## Conclusion

Built environments, such as highly safe neighbourhoods, quality green space nearby, and low local traffic were protective against high and extreme increases in weight status among children. Meanwhile, living close to shopping facilities and basic services increased the likelihood of unhealthy weight gain. However, more studies are needed to understand the influence of footpath and street conditions and access to public transport on child weight status. Ensuring children grow up in neighbourhoods with favourable built environments (e.g., highly safe, with quality green space nearby, low local traffic) is important to help them gain healthy weight.

## Supplementary information

Supplementary Tables

## Data Availability

Data are available at https://dataverse.ada.edu.au/dataverse/ncld with the permission of the Department of Social Services.
