# Supplementary material for: Association between built environments and weight status: evidence from longitudinal data of 9589 Australian children
Source: Int J Obes (Lond). 2022 May 30;46(8):1534–43. doi: 10.1038/s41366-022-01148-6 (PMC9314255; doi:10.1038/s41366-022-01148-6)
Supplement: Supplementary file 1 — Supplementary Tables [file 41366_2022_1148_MOESM1_ESM.docx]

# Supplementary Tables

**Table S1.** Perceived built environment variables

| **Variables** | **Measures of built environments from the LSAC data**  **(Responses: strongly disagree, disagree, agree, strongly agree)** |
| --- | --- |
| Safety | “This is a safe neighbourhood.” |
| Green space | “There are good parks, playgrounds and play spaces in this neighbourhood.” |
| Footpaths, roads and street lighting | “The state of footpaths, roads and street lighting is good in this neighbourhood.” |
| Public transport | “There is access to close, affordable, regular public transport in this neighbourhood.” |
| Shopping facilities | “There is access to basic shopping facilities in this neighbourhood.” |
| Basic services | “There is access to basic services such as banks, medical clinics, etc. in this neighbourhood.” |
| Heavy traffic | “There is heavy traffic on my street or road.” |

**Table S2.** BIC values of models developed

| **Variables** | **Number of trajectory groups** | **BIC values** | **Any groups with members < 5% of the total respondents** |
| --- | --- | --- | --- |
| **Built environments** | | | |
| Safety | 1 | -10770.26 | No |
|  | **2** | **-9929.62** | **No** |
|  | 3 | -9722.43 | Yes |
|  | 4 | -9757.45 | Yes |
| Green space | 1 | -21872.82 | No |
|  | 2 | -19205.88 | No |
|  | 3 | -18507.53 | No |
|  | **4** | **-18425.61** | **No** |
|  | 5 | -18440.00 | No |
| Footpaths, roads and street lighting | 1 | -28957.70 | No |
|  | 2 | -24976.48 | No |
|  | 3 | -24792.74 | No |
|  | 4 | -24610.73 | No |
|  | 5 | -24590.24 | No |
|  | **6** | **-24558.74** | **No** |
|  | 7 | -24565.81 | No |
| Public transport | 1 | -26667.73 | No |
|  | 2 | -21643.51 | No |
|  | 3 | -19953.21 | No |
|  | **4** | **-19836.26** | **No** |
|  | 5 | -19849.74 | No |
| Shopping facilities | 1 | -15605.93 | No |
|  | 2 | -13460.11 | No |
|  | 3 | -12962.91 | No |
|  | **4** | **-12942.61** | **No** |
|  | 5 | -13039.50 | No |
| Basic services | 1 | -21270.60 | No |
|  | 2 | -18615.75 | No |
|  | 3 | -17797.63 | No |
|  | **4** | **-17723.71** | **No** |
|  | 5 | -17767.77 | No |
| Heavy traffic | 1 | -26330.52 | No |
|  | 2 | -22592.52 | No |
|  | 3 | -22346.52 | No |
|  | **4** | **-22170.51** | **No** |
|  | 5 | -22179.10 | No |
| **Weight status** | | | |
| Body mass index | 1 | -119244.18 | No |
|  | 2 | -106685.23 | No |
|  | **3** | **-101123.55** | **No** |
|  | 4 | -98416.13 | Yes |
|  | 5 | -96855.45 | Yes |
| Waist circumference | 1 | -164734.94 | No |
|  | 2 | -153180.59 | No |
|  | **3** | **-148565.58** | **No** |
|  | 4 | -146707.21 | Yes |
|  | 5 | -145549.19 | Yes |

*BIC=Bayesian Information Criterion; bold=the selected model based on the lowest BIC value and no group with members < 5%*

**Table S3. Factors associated with trajectory group membership of built environment variables**

Table S3.1 Safety*

| **Variables** | **Trajectory groups** |
| --- | --- |
|  | **Group 1**  **OR (95% CI)** |
|  | ***Reference group: Group 2 – consistently in high safety*** |
| **Child characteristics** |  |
| Child’s age *(ref: 2-3 year)*  4-5 years  6-7 years  8-9 years  10-11 years  12-13 years  14-15 years | 0.93 (0.85; 1.03)  0.92 (0.83; 1.02)  **0.88 (0.80; 0.98)**  **0.87 (0.79; 0.97)**  **0.89 (0.80; 0.99)**  **0.85 (0.75; 0.97)** |
| Child’s sex *(ref: Female)*  Male | 1.04 (0.96; 1.12) |
| Child Indigenous status *(ref: Indigenous)*  Not Indigenous | **1.68 (1.37; 2.04)** |
| Child speaks a language other than English *(ref: Yes)*  No | 1.07 (0.95; 1.22) |
| **Family characteristics** |  |
| Caregiver education *(ref:* ≤ High school)  > High school | **1.12 (1.01; 1.25)** |
| Family weekly income (in thousands) | **1.10 (1.07; 1.13)** |
| Family structure *(ref: One-caregiver family)*  Two-caregiver family | **1.42 (1.29; 1.56)** |
| Number of siblings, mean (SD) | **0.96 (0.93; 0.99)** |
| **Neighbourhood characteristics** |  |
| Area disadvantage (SEIFA) *(ref: High)*  Moderate  Low | **1.31 (1.22; 1.41)**  **2.07 (1.89; 2.27)** |
| Area accessibility (ARIA) *(ref: Highly accessible)*  Accessible  Moderately accessible  Remote  Very remote | **1.14 (1.04; 1.25)**  **1.45 (1.30; 1.62)**  1.12 (0.88; 1.41)  1.07 (0.80; 1.43) |

*OR= adjusted odds ratio; CI=confidence interval; bold=p-value<0.05*

** The analysis was conducted using multilevel binary logistic regression.*

Table S3.2 Green space quality

| **Variables** | **Trajectory groups** | | |
| --- | --- | --- | --- |
|  | **Group 1**  **RRR (95% CI)** | **Group 2**  **RRR (95% CI)** | **Group 3**  **RRR (95% CI)** |
|  | ***Reference group: Group 4 – consistently in low quality*** | | |
| **Child characteristics** |  |  |  |
| Child’s age *(ref: 2-3 year)*  4-5 years  6-7 years  8-9 years  10-11 years  12-13 years  14-15 years | 0.93 (0.82; 1.04)  1.04 (0.92; 1.17)  1.09 (0.97; 1.23)  1.09 (0.97; 1.24)  1.07 (0.93; 1.22)  1.08 (0.93; 1.25) | 0.85 (0.72; 1.01)  0.99 (0.83; 1.17)  1.04 (0.88; 1.25)  1.07 (0.89; 1.27)  1.09 (0.90; 1.32)  0.97 (0.78; 1.21) | **0.86 (0.78; 0.94)**  0.96 (0.86; 1.04)  0.95 (0.87; 1.05)  0.94 (0.86; 1.04)  0.93 (0.84; 1.04)  0.89 (0.79; 1.01) |
| Child’s sex *(ref: Female)*  Male | **0.91 (0.83; 0.99)** | 0.96 (0.84; 1.09) | 0.93 (0.87; 1.00) |
| Child Indigenous status *(ref: Indigenous)*  Not Indigenous | **1.50 (1.19; 1.91)** | 0.85 (0.62; 1.17) | **1.38 (1.14; 1.66)** |
| Child speaks a language other than English *(ref: Yes)*  No | 0.87 (0.75; 1.01) | 0.91 (0.73; 1.13) | 0.98 (0.87; 1.11) |
| **Family characteristics** |  |  |  |
| Caregiver education *(ref:* ≤ High school)  > High school | 1.06 (0.93; 1.19) | 1.16 (0.97; 1.39) | **1.32 (1.20; 1.46)** |
| Family weekly income (in thousands) | 1.01 (0.97; 1.04) | 1.03 (0.99; 1.08) | **1.11 (1.08; 1.14)** |
| Family structure *(ref: One-caregiver family)*  Two-caregiver family | 0.93 (0.84; 1.05) | 0.90 (0.77; 1.07) | 0.91 (0.83; 1.00) |
| Number of siblings, mean (SD) | 1.02 (0.98; 1.06) | 0.98 (0.92; 1.03) | 0.95 (0.94; 1.00) |
| **Neighbourhood characteristics** |  |  |  |
| Area disadvantage (SEIFA) *(ref: High)*  Moderate  Low | **1.17 (1.07; 1.27)**  **1.28 (1.15; 1.43)** | **1.34 (1.18; 1.52)**  **1.58 (1.35; 1.85)** | **1.54 (1.44; 1.65)**  **2.40 (2.20; 2.62)** |
| Area accessibility (ARIA) *(ref: Highly accessible)*  Accessible  Moderately accessible  Remote  Very remote | 0.93 (0.84; 1.04)  **0.79 (0.70; 0.90)**  1.09 (0.84; 1.41)  1.11 (0.81; 1.51) | 0.86 (0.74; 1.01)  **0.62 (0.52; 0.75)**  0.84 (0.57; 1.23)  0.85 (0.53; 1.35) | **0.40 (0.37; 0.44)**  **0.28 (0.26; 0.31)**  **0.32 (0.26; 0.40)**  **0.28 (0.22; 0.37)** |

*RRR= adjusted relative-risk ratio; CI=confidence interval; bold=p-value<0.05*

Table S3.3 Footpaths, roads and street lighting

| **Variables** | **Trajectory groups** | | | | |
| --- | --- | --- | --- | --- | --- |
|  | **Group 2**  **RRR (95% CI)** | **Group 3**  **RRR (95% CI)** | **Group 4**  **RRR (95% CI)** | **Group 5**  **RRR (95% CI)** | **Group 6**  **RRR (95% CI)** |
|  | ***Reference group: Group 1 – consistently in low quality*** | | | | |
| **Child characteristics** |  |  |  |  |  |
| Child’s age *(ref: 2-3 year)*  4-5 years  6-7 years  8-9 years  10-11 years  12-13 years  14-15 years | 0.94 (0.83; 1.07)  0.97 (0.85; 1.10)  0.97 (0.85; 1.11)  1.01 (0.89; 1.16)  1.05 (0.91; 1.22)  0.95 (0.81; 1.12) | **0.81 (0.75; 0.88)**  **0.80 (0.74; 0.87)**  **0.79 (0.72; 0.84)**  **0.78 (0.71; 0.84)**  **0.80 (0.73; 0.87)**  **0.65 (0.59; 0.72)** | 0.99 (0.86; 1.15)  1.01 (0.87; 1.17)  1.00 (0.86; 1.16)  0.99 (0.85; 1.16)  1.00 (0.84; 1.18)  0.89 (0.74; 1.08) | 1.10 (1.00; 1.21)  1.01 (0.92; 1.11)  0.97 (0.88; 1.07)  0.97 (0.88; 1.07)  1.01 (0.92; 1.12)  0.96 (0.86; 1.08) | **0.84 (0.73; 0.96)**  **0.85 (0.74; 0.98)**  **0.85 (0.74; 0.98)**  **0.83 (0.72; 0.96)**  **0.83 (0.70; 0.97)**  **0.71 (0.59; 0.85**) |
| Child’s sex *(ref: Female)*  Male | **0.85 (0.77; 0.93)** | **0.88 (0.83; 0.94)** | **0.88 (0.79; 0.99)** | **0.92 (0.88; 0.96)** | **0.84 (0.76; 0.94)** |
| Child Indigenous status *(ref: Indigenous)*  Not Indigenous | 0.99 (0.76; 1.28) | 1.10 (0.93; 1.29) | 0.85 (0.62; 1.16) | 0.95 (0.84; 1.07) | 0.86 (0.64; 1.15) |
| Child speaks a language other than English *(ref: Yes)*  No | **0.76 (0.65; 0.90)** | **0.48 (0.44; 0.53)** | **0.56 (0.46; 0.67)** | **0.66 (0.61; 0.72)** | **0.53 (0.44; 0.63)** |
| **Family characteristics** |  |  |  |  |  |
| Caregiver education *(ref:* ≤ High school)  > High school | 0.93 (0.81; 1.06) | 0.95 (0.88; 1.04) | 1.05 (0.89; 1.24) | 1.03 (0.96; 1.11) | 1.06 (0.90; 1.24) |
| Family weekly income (in thousands) | 0.97 (0.93; 1.00) | **1.05 (1.03; 1.07)** | 1.02 (0.99; 1.06) | 1.02 (1.00; 1.04) | **1.05 (1.01; 1.08)** |
| Family structure *(ref: One-caregiver family)*  Two-caregiver family | 0.89 (0.79; 1.00) | **0.80 (0.74; 0.86)** | **0.75 (0.65; 0.86)** | **0.71 (0.66; 0.76)** | **0.72 (0.63; 0.82)** |
| Number of siblings, mean (SD) | 0.99 (0.95; 1.03) | **0.96 (0.94; 0.99)** | **0.94 (0.90; 0.99)** | 1.00 (0.98; 1.03) | 1.02 (0.98; 1.07) |
| **Neighbourhood characteristics** |  |  |  |  |  |
| Area disadvantage (SEIFA) *(ref: High)*  Moderate  Low | 1.07 (0.97; 1.17)  1.07 (0.95; 1.19) | **1.23 (1.16; 1.30)**  **1.52 (1.42; 1.63)** | **1.23 (1.11; 1.39)**  **1.42 (1.24; 1.62)** | 0.95 (0.90; 1.01)  0.96 (0.90; 1.02) | **1.15 (1.03; 1.28)**  **1.24 (1.09; 1.41)** |
| Area accessibility (ARIA) *(ref: Highly accessible)*  Accessible  Moderately accessible  Remote  Very remote | **0.59 (0.53; 0.66)**  **0.53 (0.46; 0.60)**  **0.48 (0.35; 0.64)**  1.11 (0.79; 1.56) | **0.36 (0.33; 0.38)**  **0.19 (0.18; 0.21)**  **0.19 (0.15; 0.23)**  **0.27 (0.21; 0.35)** | **0.38 (0.33; 0.44)**  **0.29 (0.24; 0.34)**  **0.34 (0.24; 0.49)**  **0.52 (0.33; 0.81)** | **0.60 (0.57; 0.64)**  **0.55 (0.51; 0.58)**  **0.53 (0.46; 0.61)**  0.95 (0.81; 1.13) | **0.39 (0.34; 0.45)**  **0.29 (0.25; 0.34)**  **0.35 (0.25; 0.49)**  **0.56 (0.37; 0.85)** |

*RRR= adjusted relative-risk ratio; CI=confidence interval; bold=p-value<0.05*

Table S3.4 Public transport

| **Variables** | **Trajectory groups** | | |
| --- | --- | --- | --- |
|  | **Group 1**  **RRR (95% CI)** | **Group 2**  **RRR (95% CI)** | **Group 3**  **RRR (95% CI)** |
|  | ***Reference group: Group 4 – consistently in low access*** | | |
| **Child characteristics** |  |  |  |
| Child’s age *(ref: 2-3 year)*  4-5 years  6-7 years  8-9 years  10-11 years  12-13 years  14-15 years | 1.00 (0.87; 1.14)  1.07 (0.94; 1.24)  1.12 (0.97; 1.29)  1.13 (0.98; 1.30)  1.15 (0.99; 1.33)  1.06 (0.89; 1.26) | **1.19 (1.04; 1.35)**  **1.30 (1.14; 1.40)**  **1.39 (1.21; 1.58)**  **1.43 (1.25; 1.64)**  **1.44 (1.24; 1.66)**  **1.58 (1.34; 1.86)** | 0.94 (0.86; 1.02)  0.99 (0.91; 1.08)  1.00 (0.92; 1.09)  1.00 (0.92; 1.10)  1.01 (0.91; 1.11)  0.94 (0.84; 1.05) |
| Child’s sex *(ref: Female)*  Male | **1.15 (1.04; 1.27)** | **1.14 (1.04; 1.25)** | **1.08 (1.02; 1.16)** |
| Child Indigenous status *(ref: Indigenous)*  Not Indigenous | **0.64 (0.49; 0.85)** | **0.62 (0.48; 0.80)** | **0.48 (0.40; 0.58)** |
| Child speaks a language other than English *(ref: Yes)*  No | **0.59 (0.50; 0.71)** | **0.76 (0.64; 0.89)** | **0.55 (0.49; 0.61)** |
| **Family characteristics** |  |  |  |
| Caregiver education *(ref:* ≤ High school)  > High school | 1.02 (0.87; 1.19) | **0.84 (0.74; 0.96)** | **0.82 (0.74; 0.80)** |
| Family weekly income (in thousands) | **1.04 (1.01; 1.07)** | **0.94 (0.91; 0.98)** | **1.11 (1.08; 1.14)** |
| Family structure *(ref: One-caregiver family)*  Two-caregiver family | **0.72 (0.63; 0.82)** | 0.91 (0.81; 1.03) | **0.78 (0.72; 0.85)** |
| Number of siblings, mean (SD) | **0.94 (0.89; 0.98)** | 1.03 (0.99; 1.07) | 0.98 (0.95; 1.01) |
| **Neighbourhood characteristics** |  |  |  |
| Area disadvantage (SEIFA) *(ref: High)*  Moderate  Low | 1.04 (0.94; 1.15)  1.03 (0.92; 1.17) | 0.97 (0.89; 1.07)  0.97 (0.86; 1.08) | 1.06 (0.99; 1.13)  **1.10 (1.02; 1.19)** |
| Area accessibility (ARIA) *(ref: Highly accessible)*  Accessible  Moderately accessible  Remote  Very remote | **0.44 (0.39; 0.50)**  **0.30 (0.26; 0.34)**  **0.23 (0.17; 0.31)**  **0.31 (0.21; 0.44)** | **0.32 (0.29; 0.36)**  **0.17 (0.15; 0.20)**  **0.13 (0.09; 0.17)**  **0.08 (0.06; 0.13)** | **0.15 (0.14; 0.16)**  **0.05 (0.05; 0.06)**  **0.04 (0.03; 0.05)**  **0.02 (0.01; 0.02)** |

*RRR= adjusted relative-risk ratio; CI=confidence interval; bold=p-value<0.05*

Table S3.5 Shopping facilities

| **Variables** | **Trajectory groups** | | |
| --- | --- | --- | --- |
|  | **Group 1**  **RRR (95% CI)** | **Group 2**  **RRR (95% CI)** | **Group 4**  **RRR (95% CI)** |
|  | ***Reference group: Group 3 – consistently in low access*** | | |
| **Child characteristics** |  |  |  |
| Child’s age *(ref: 2-3 year)*  4-5 years  6-7 years  8-9 years  10-11 years  12-13 years  14-15 years | 0.96 (0.84; 1.09)  1.04 (0.91; 1.19)  1.05 (0.92; 1.21)  1.06 (0.92; 1.22)  1.07 (0.92; 1.24)  1.05 (0.88; 1.24) | 0.96 (0.87; 1.05)  1.02 (0.93; 1.12)  1.01 (0.92; 1.11)  1.00 (0.91; 1.10)  1.01 (0.91; 1.12)  0.99 (0.88; 1.11) | 1.04 (0.92; 1.18)  1.13 (1.00; 1.29)  **1.17 (1.03; 1.33)**  **1.19 (1.04; 1.35)**  **1.19 (1.03; 1.36)**  **1.21 (1.04; 1.42)** |
| Child’s sex *(ref: Female)*  Male | **0.85 (0.77; 0.94)** | **0.88 (0.82; 0.94)** | 0.93 (0.85; 1.02) |
| Child Indigenous status *(ref: Indigenous)*  Not Indigenous | 1.22 (0.93; 1.60) | 1.06 (0.88; 1.28) | 1.05 (0.82; 1.34) |
| Child speaks a language other than English *(ref: Yes)*  No | **0.65 (0.55; 0.77)** | 0.95 (0.84; 1.06) | **0.76 (0.65; 0.89)** |
| **Family characteristics** |  |  |  |
| Caregiver education *(ref:* ≤ High school)  > High school | 1.10 (0.95; 1.27) | 1.07 (0.97; 1.18) | 0.99 (0.87; 1.13) |
| Family weekly income (in thousands) | 1.01 (0.98; 1.05) | **1.07 (1.05; 1.10)** | 1.02 (1.00; 1.06) |
| Family structure *(ref: One-caregiver family)*  Two-caregiver family | **0.69 (0.61; 0.79)** | **0.76 (0.70; 0.83)** | **0.81 (0.71; 0.91)** |
| Number of siblings, mean (SD) | **0.91 (0.87 0.95)** | **0.88 (0.85; 0.91)** | **0.91 (0.87; 0.95)** |
| **Neighbourhood characteristics** |  |  |  |
| Area disadvantage (SEIFA) *(ref: High)*  Moderate  Low | **1.13 (1.03; 1.24)**  1.11 (0.99; 1.26) | **1.20 (1.12; 1.28)**  **1.37 (1.26; 1.50)** | 0.98 (0.90; 1.07)  0.89 (0.80; 1.00) |
| Area accessibility (ARIA) *(ref: Highly accessible)*  Accessible  Moderately accessible  Remote  Very remote | **0.38 (0.33; 0.42)**  **0.30 (0.26; 0.34)**  **0.35 (0.26; 0.47)**  **0.27 (0.19; 0.39)** | **0.13 (0.12; 0.15)**  **0.08 (0.07; 0.09)**  **0.10 (0.08; 0.12)**  **0.05 (0.04; 0.07)** | **0.35 (0.32; 0.40)**  **0.31 (0.27; 0.35)**  **0.39 (0.30; 0.51)**  **0.19 (0.13; 0.26)** |

*RRR= adjusted relative-risk ratio; CI=confidence interval; bold=p-value<0.05*

Table S3.6 Basic services

| **Variables** | **Trajectory groups** | | |
| --- | --- | --- | --- |
|  | **Group 1**  **RRR (95% CI)** | **Group 2**  **RRR (95% CI)** | **Group 3**  **RRR (95% CI)** |
|  | ***Reference group: Group 4 – consistently in low access*** | | |
| **Child characteristics** |  |  |  |
| Child’s age *(ref: 2-3 year)*  4-5 years  6-7 years  8-9 years  10-11 years  12-13 years  14-15 years | 1.03 (0.87; 1.22)  1.02 (0.86; 1.21)  1.03 (0.86; 1.23)  1.00 (0.83; 1.20)  0.92 (0.76; 1.12)  0.91 (0.73; 1.14) | 1.04 (0.92; 1.17)  0.99 (0.87; 1.11)  0.98 (0.87; 1.11)  0.99 (0.87; 1.12)  0.95 (0.83; 1.09)  0.90 (0.77; 1.05) | 0.93 (0.85; 1.02)  0.91 (0.82; 1.00)  **0.88 (0.80; 0.98)**  **0.87 (0.78; 0.96)**  **0.83 (0.75; 0.93)**  **0.76 (0.68; 0.87)** |
| Child’s sex *(ref: Female)*  Male | 0.94 (0.83; 1.07) | 1.00 (0.91; 1.10) | 0.99 (0.92; 1.07) |
| Child Indigenous status *(ref: Indigenous)*  Not Indigenous | 1.03 (0.73; 1.45) | **0.65 (0.52; 0.82)** | 1.14 (0.94; 1.38) |
| Child speaks a language other than English *(ref: Yes)*  No | **0.57 (0.45; 0.72)** | **0.43 (0.37; 0.50)** | **0.56 (0.50; 0.64)** |
| **Family characteristics** |  |  |  |
| Caregiver education *(ref:* ≤ High school)  > High school | 1.13 (0.94; 1.36) | **1.17 (1.02; 1.33)** | **1.16 (1.05; 1.29)** |
| Family weekly income (in thousands) | 1.04 (0.99; 1.10) | **1.04 (1.01; 1.08)** | **1.13 (1.10; 1.16)** |
| Family structure *(ref: One-caregiver family)*  Two-caregiver family | **0.73 (0.62; 0.87)** | **0.69 (0.61; 0.77)** | **0.69 (0.63; 0.75)** |
| Number of siblings, mean (SD) | 1.00 (0.95; 1.06) | **0.94 (0.90; 0.98)** | **0.92 (0.90; 0.95)** |
| **Neighbourhood characteristics** |  |  |  |
| Area disadvantage (SEIFA) *(ref: High)*  Moderate  Low | 0.94 (0.83; 1.07)  0.93 (0.79; 1.09) | 0.97 (0.89; 1.05)  0.92 (0.83; 1.03) | 1.05 (0.98; 1.13)  **1.25 (1.14; 1.36)** |
| Area accessibility (ARIA) *(ref: Highly accessible)*  Accessible  Moderately accessible  Remote  Very remote | **0.59 (0.50; 0.69)**  **0.54 (0.45; 0.65)**  **0.52 (0.36; 0.76)**  **0.51 (0.32; 0.81)** | **0.57 (0.51; 0.63)**  **0.44 (0.29; 0.50)**  **0.35 (0.27; 0.47)**  **0.33 (0.24; 0.47)** | **0.22 (0.20; 0.24)**  **0.15 (0.13; 0.16)**  **0.13 (0.10; 0.16)**  **0.14 (0.11; 0.19)** |

*RRR= adjusted relative-risk ratio; CI=confidence interval; bold=p-value<0.05*

Table S3.7 Heavy traffic

| **Variables** | **Trajectory groups** | | |
| --- | --- | --- | --- |
|  | **Group 2**  **RRR (95% CI)** | **Group 3**  **RRR (95% CI)** | **Group 4**  **RRR (95% CI)** |
|  | ***Reference group: Group 1 – consistently in low traffic*** | | |
| **Child characteristics** |  |  |  |
| Child’s age *(ref: 2-3 year)*  4-5 years  6-7 years  8-9 years  10-11 years  12-13 years  14-15 years | 1.03 (0.92; 1.14)  1.06 (0.95; 1.18)  1.09 (0.98; 1.22)  1.10 (0.98; 1.24)  1.12 (1.00; 1.27)  1.08 (0.94; 1.24) | **1.13 (1.01; 1.26)**  1.08 (0.96; 1.21)  1.10 (0.98; 1.23)  1.10 (0.98; 1.24)  1.07 (0.94; 1.22)  1.14 (0.99; 1.32) | 1.06 (0.90; 1.17)  1.06 (0.93; 1.20)  1.08 (0.94; 1.23)  1.08 (0.94; 1.23)  1.12 (0.97; 1.29)  1.06 (0.89; 1.25) |
| Child’s sex *(ref: Female)*  Male | 0.92 (0.85; 1.00) | **1.11 (1.02; 1.20)** | 1.02 (0.92; 1.12) |
| Child Indigenous status *(ref: Indigenous)*  Not Indigenous | **0.74 (0.60; 0.92)** | **0.71 (0.57; 0.88)** | **0.69 (0.53; 0.91)** |
| Child speaks a language other than English *(ref: Yes)*  No | 0.91 (0.80; 1.05) | **0.86 (0.75; 0.99)** | 0.91 (0.77; 1.07) |
| **Family characteristics** |  |  |  |
| Caregiver education *(ref:* ≤ High school)  > High school | **0.82 (0.73; 0.92)** | **0.83 (0.74; 0.94)** | 1.01 (0.87; 1.16) |
| Family weekly income (in thousands) | 0.97 (0.95; 1.00) | **0.91 (0.88; 0.94)** | **0.94 (0.91; 0.97)** |
| Family structure *(ref: One-caregiver family)*  Two-caregiver family | **0.83 (0.74; 0.92)** | **0.80 (0.71; 0.88)** | **0.88 (0.77; 0.99)** |
| Number of siblings, mean (SD) | 1.02 (0.72; 1.46) | 1.05 (1.00; 1.08) | 1.04 (0.99; 1.08) |
| **Neighbourhood characteristics** |  |  |  |
| Area disadvantage (SEIFA) *(ref: High)*  Moderate  Low | **0.89 (0.82; 0.96)**  **0.82 (0.74; 0.90)** | **0.88 (0.81; 0.96)**  **0.80 (0.72; 0.88)** | **0.82 (0.74; 0.90)**  **0.77 (0.69; 0.87)** |
| Area accessibility (ARIA) *(ref: Highly accessible)*  Accessible  Moderately accessible  Remote  Very remote | 0.91 (0.83; 1.01)  1.07 (0.95; 1.21)  0.91 (0.70; 1.18)  0.83 (0.60; 1.15) | 1.02 (0.92; 1.13)  1.10 (0.97; 1.24)  1.02 (0.78; 1.33)  1.07 (0.78; 1.48) | **0.81 (0.72; 0.92)**  0.99 (0.85; 1.14)  **0.66 (0.47; 0.92)**  **0.59 (0.39; 0.90)** |

*RRR= adjusted relative-risk ratio; CI=confidence interval; bold=p-value<0.05*
